# Author Correction: Decision uncertainty as a context for motor memory

**DOI:** 10.1038/s41562-024-01960-2

**Published:** 2024-07-23

**Authors:** Kisho Ogasa, Atsushi Yokoi, Gouki Okazawa, Morimichi Nishigaki, Masaya Hirashima, Nobuhiro Hagura

**Affiliations:** 1 Center for Information and Neural Networks (CiNet), National Institute of Information and Communications Technology (NICT), Osaka, Japan; 2 Graduate School of Frontier Biosciences, Osaka University, Osaka, Japan; 3 Institute of Neuroscience, Key Laboratory of Primate Neurobiology, Center for Excellence in Brain Science and Intelligence Technology, Chinese Academy of Sciences, Shanghai, China; 4 Innovative Research Excellence, Honda R&D Co. Ltd, Utsunomiya, Japan

**Keywords:** Decision, Human behaviour, Human behaviour

Correction to: *Nature Human Behaviour* 10.1038/s41562-024-01911-x, published online 11 June 2024.

This article was originally published under standard Springer Nature license (© The Author(s), under exclusive licence to Springer Nature Limited). It is now available as an open-access paper under a Creative Commons Attribution 4.0 International license, © The Author(s). The error has been corrected in the HTML and PDF versions of the article.

