# Supplementary material for: Decision uncertainty as a context for motor memory
Source: Nat Hum Behav. 2024 Jun 11;8(9):1738–51. doi: 10.1038/s41562-024-01911-x (PMC11420082; doi:10.1038/s41562-024-01911-x)
Supplement: Supplementary file 1 — Supplementary Figs. 1–4, with results and discussion in the legends. [file 41562_2024_1911_MOESM1_ESM.pdf]

nature human behaviour

Supplementary information

https://doi.org/10.1038/s41562-024-01911-x

Decision uncertainty as a context for motor memory

In the format provided by the authors and unedited

Supplementary Materials

Supplementary Figures & Results

Figure. S1. Choice and reaction time data in the test phase of Experiment 1.
Figure S2. Results of the dual force-field learning in Experiments 2 and 3.
Figure S3. Result of Experiment 4.
Figure S4. Force profile during the probe trials across different experiments.

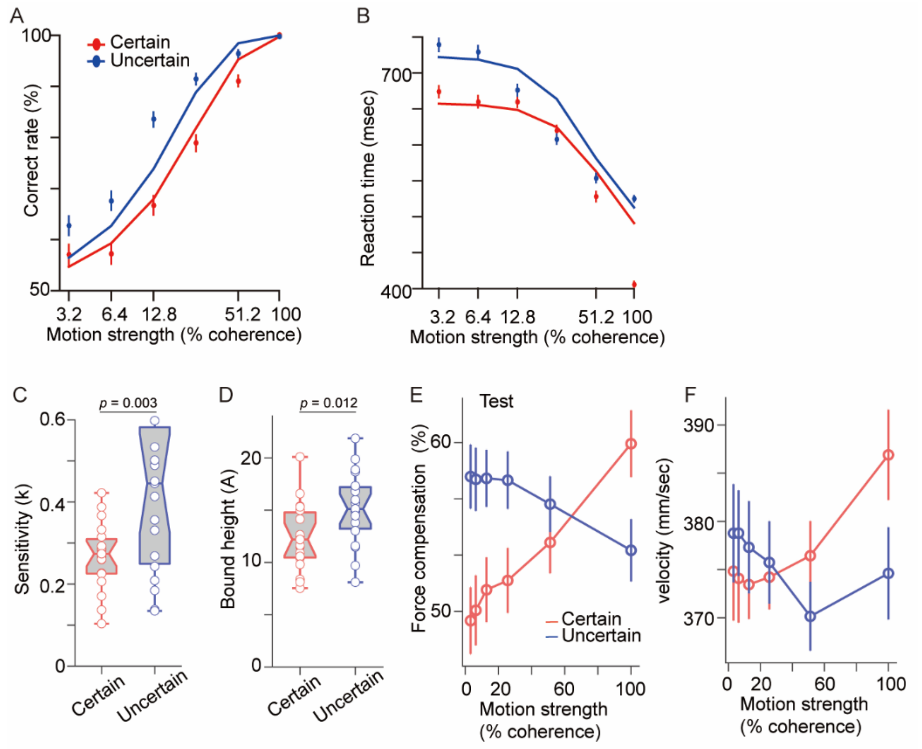

Figure. S1. Choice and reaction time data in the test phase of Experiment 1.

A, B: Correct rate (**A**) and reaction time (**B**) plotted against different motion coherence levels (n=19 for both certain and uncertain group). A typical psychometric and chronometric function for the random-dot-motion direction decision was observed for both participant groups. The fitted line was derived from the drift-diffusion model parameters applied to the data (see Materials and Methods). **C, D:** Sensitivity to the decision evidence (**C**) and the height of the evidence accumulation bound (**D**) for each certain-decision and uncertain-decision group, as estimated from the drift-diffusion model. The uncertain-decision group had significantly higher bound height (**D**), probably because the participants in this group became more cautious in their decision due to repeated exposure to the difficult stimulus during the learning phase. It is likely that difficult stimuli also facilitated perceptual learning in this group and improved their visual motion sensitivity *k* (**C**) (two-sided paired t-test). **E**: The force compensation level across different uncertainty levels in each group, which is the base data of the generalization data presented in the main Figure 1**F**. **F**: Peak velocity of the movement across different uncertainty levels in each group. In the certain-decision group, there was a slight tendency to increase the speed towards the more certain visual stimulus (100% coherent motion) (approximately 3% increase in speed from that of action following 3.2% coherent motion decision), but this trend was not observed in the uncertain-decision group (ANOVA interaction effect; $F(5,180)=3.56$), $p= 0.004$, $\eta 2=0.09$). This possibly reflects the slight difference in the decision parameters between the two groups, after the repeated exposure to the same visual stimuli in the learning phase. Error bars and shading indicate the standard error of the mean across participants. In the boxplots, each dot represents a participant, while the midline of the box represents the median of the data. The box itself spans from the 25th to the 75th percentile, and the whiskers show the range (min to max) of the data. Outliers are determined by data points that are greater than [1.5 × size from the box]. *: $p<0.05$.

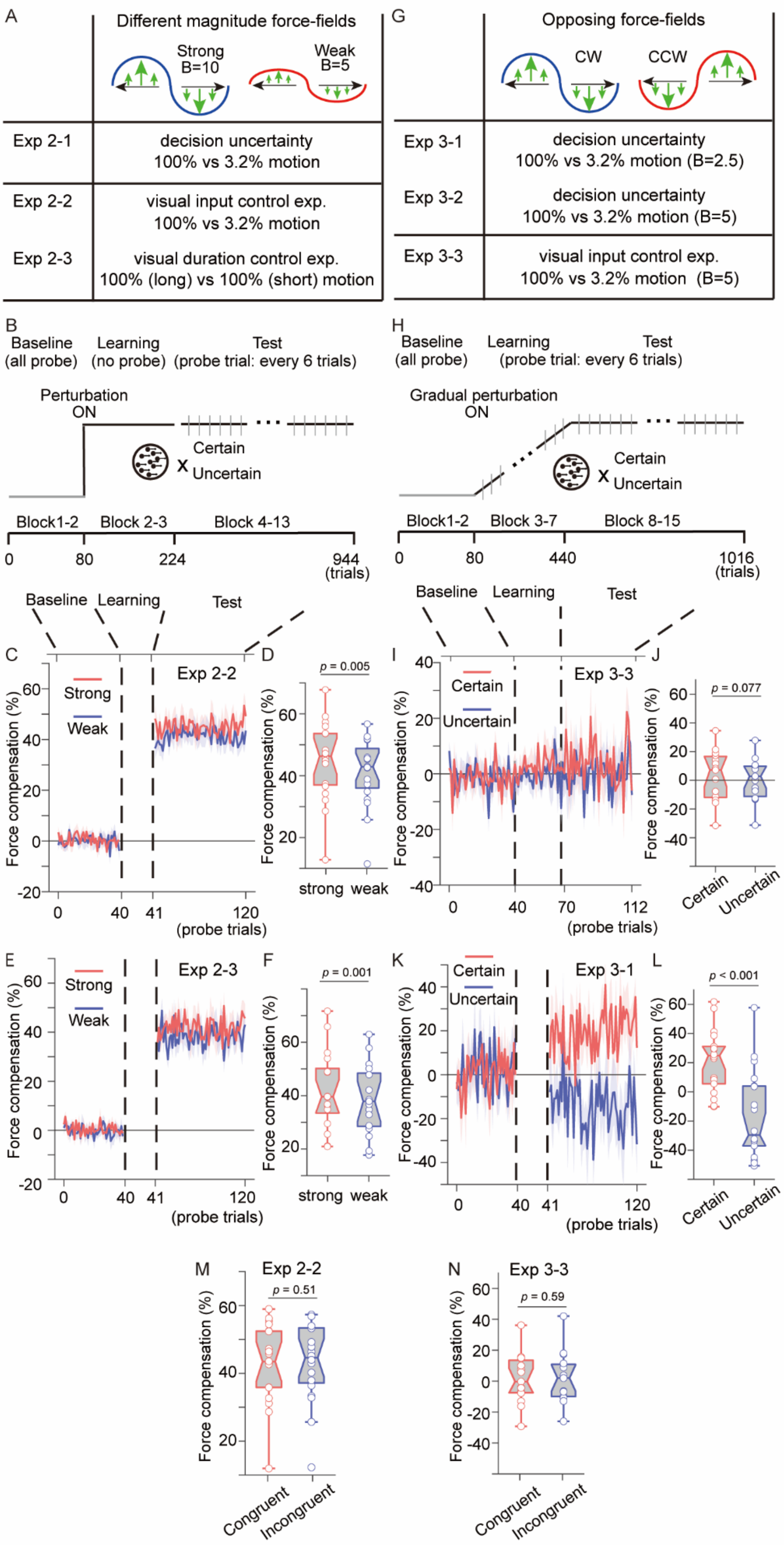

A
Different magnitude force-fields
Strong B=10
Weak B=5
Exp 2-1
decision uncertainty
100% vs 3.2% motion
Exp 2-2
visual input control exp.
100% vs 3.2% motion
Exp 2-3
visual duration control exp.
100% (long) vs 100% (short) motion
G
Opposing force-fields
CW
CCW
Exp 3-1
decision uncertainty
100% vs 3.2% motion (B=2.5)
Exp 3-2
decision uncertainty
100% vs 3.2% motion (B=5)
Exp 3-3
visual input control exp.
100% vs 3.2% motion (B=5)
B
Baseline (all probe)
Learning (no probe)
Test (probe trial: every 6 trials)
Perturbation ON
Certain
Uncertain
Block1-2
Block 2-3
Block 4-13
0
80
224
944 (trials)
H
Baseline (all probe)
Learning
Test (probe trial: every 6 trials)
Gradual perturbation ON
Certain
Uncertain
Block1-2
Block 3-7
Block 8-15
0
80
440
1016 (trials)
Baseline
Learning
Test
C
Exp 2-2
Strong
Weak
Force compensation (%)
(probe trials)
D
p = 0.005
strong
weak
I
Exp 3-3
Certain
Uncertain
(probe trials)
J
p = 0.077
Certain
Uncertain
E
Exp 2-3
Strong
Weak
(probe trials)
F
p = 0.001
strong
weak
K
Exp 3-1
Certain
Uncertain
(probe trials)
L
p < 0.001
Certain
Uncertain
M
Exp 2-2
p = 0.51
Congruent
Incongruent
N
Exp 3-3
p = 0.59
Congruent
Incongruent

Figure S2. Results of the dual force-field learning in Experiments 2 and 3.

A, G: Conditions for Experiments 2 (**A**) and 3 (**G**). **B, H**: Trial structures from Experiments 2 and Experiment 3-1 (**B**), and Experiment 3-3 (**G**).

C, D, E, F: Results of the visual control (Experiment 2-2 (n=20); **C, D**) and duration control experiments (Experiment 2-3 (n=17); **E, F**) in Experiment 2. Significant differences were observed in the force compensation level between the strong and weak conditions in both experiments (Experiment 2-2: two sided paired t-test, t[19]=3.21, *p*=0.005, dz=0.71, Experiment 2-3: two sided paired t-test, t[16]=3.92, *p*=0.001, dz=0.95). However, the magnitude of the effect was significantly smaller than in Experiment 2-1 in which the two force fields were associated with different decision uncertainty levels. See also Figure 2 in the main test and Materials and Methods for details of the experiments.

I, J, K, L: Results of the opposing force field (Experiment 3-1 (n=18); B=±2.5 (N/[ms-1]) (**K, L**) and the visual control experiment (Experiment 3-3 (n=15)) (**I, J**) in Experiment 3. Participants successfully learned to separate the different force fields associated with different decision uncertainty levels in Experiment 3-1 (**K, L)** (two sided paired t-test, t[17]=5.91, *p*=1.70×10-5, dz=1.4), but were unable to do so in the condition where participants saw the same visual stimulus without making any direction decisions (Experiment 3-3; **I, J**) (two sided paired t-test, t[14]=1.91, *p*=0.08, dz=0.49). See also Figure 3 in the main test and Materials and Methods for details of the experiments.

M, N: The effect of stimulus-action congruency on force compensation levels in visual control experiments. Trials were classified according to whether the directions of visual motion and movement matched (congruent) or not (incongruent). In both Experiments 2-2 (**M**) and 3-3 (**N**), force compensation level was not affected by the congruency effect (Experiment 2-2; two sided paired t-test, t[19]=0.51, *p*=0.62, dz=0.11, Experiment 3-3; paired t-test, t[14]=0.55, *p*=0.59, dz=0.14).

Error bars and shading indicate standard error of the mean across participants. In the boxplots, each dot represents a participant, and the midline of the box represents the median of the data. The box itself spans from the 25th to the 75th percentile, and the whiskers show the range (min to max) of the data. Outliers are determined by data points that are greater than [1.5 x size from the box].

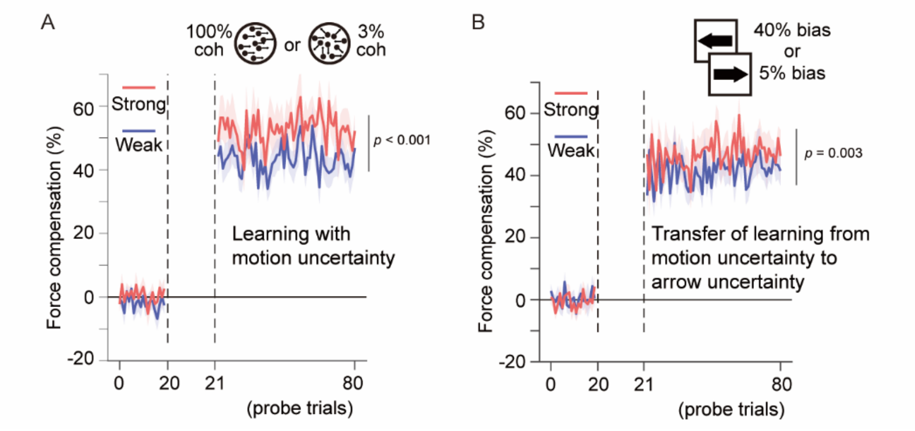

Figure S3. Result of Experiment 4.

Progression of the force field learning in Experiment 4. Force-field learning while making the direction decision for a random-dot motion stimulus (**A**), and while making the direction decision for an arrow sequence (**B**) (two-sided paired t-test). Note that for the arrow sequence stimuli, participants never made a decision in association with any type of force field (see Figure 4 in the main text). Therefore, any difference in force compensation between different arrow decision uncertainty levels in the test phase was necessary because of the association between decision uncertainty and force field strength learned through random-dot motion stimuli. Shading indicates the standard error of the mean across participants.

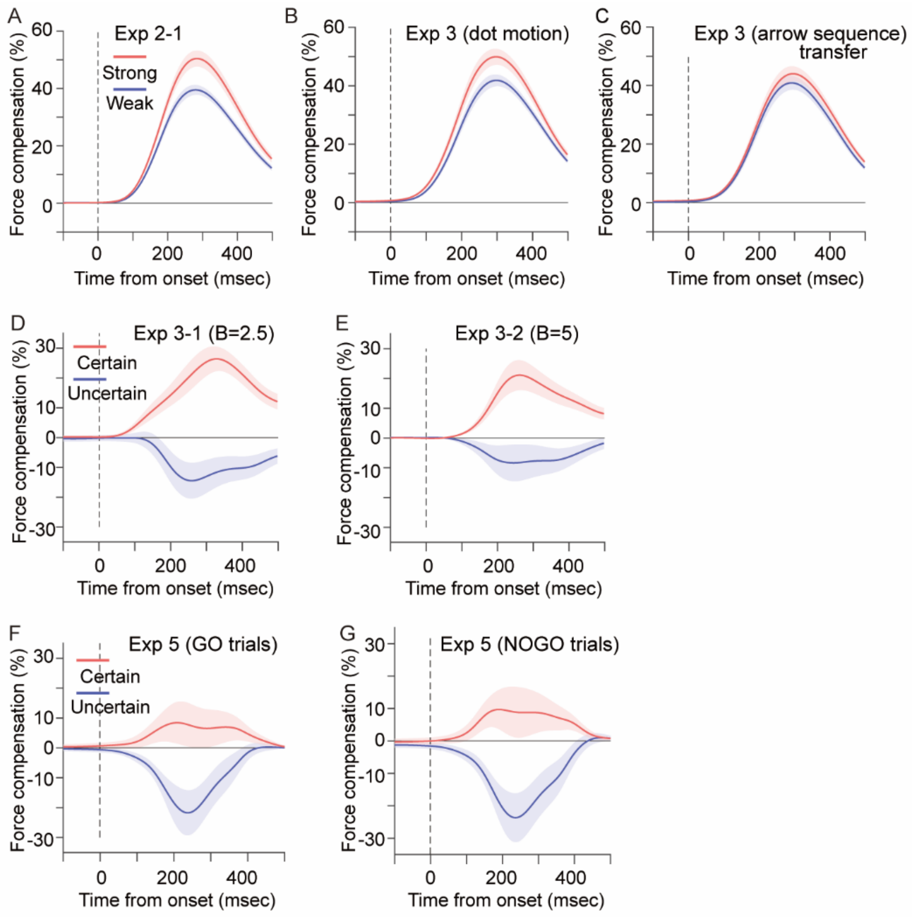

Figure S4. Force profile during the probe trials across different experiments.

The resisting force (N) in each probe trial was normalized by the velocity peak of each trial, and then divided by the force field strength to calculate the percentage of ideal force of that trial (force compensation). Data were averaged across the participants. Shading indicates the standard error of the mean across participants.
