# Supplementary material for: Decision uncertainty as a context for motor memory
Source: Nat Hum Behav. 2024 Jun 11;8(9):1738–51. doi: 10.1038/s41562-024-01911-x (PMC11420082; doi:10.1038/s41562-024-01911-x)
Supplement: Supplementary file 2 — Reporting Summary [file 41562_2024_1911_MOESM2_ESM.pdf]

nature portfolio

Corresponding author(s): Nobuhiro Hagura

Last updated by author(s): May 5, 2024

Reporting Summary

Nature Portfolio wishes to improve the reproducibility of the work that we publish. This form provides structure for consistency and transparency in reporting. For further information on Nature Portfolio policies, see our Editorial Policies and the Editorial Policy Checklist.

Statistics

For all statistical analyses, confirm that the following items are present in the figure legend, table legend, main text, or Methods section.

n/a	Confirmed	
☐	☒	The exact sample size (*n*) for each experimental group/condition, given as a discrete number and unit of measurement
☐	☒	A statement on whether measurements were taken from distinct samples or whether the same sample was measured repeatedly
☐	☒	The statistical test(s) used AND whether they are one- or two-sided<br>*Only common tests should be described solely by name; describe more complex techniques in the Methods section.*
☐	☒	A description of all covariates tested
☐	☒	A description of any assumptions or corrections, such as tests of normality and adjustment for multiple comparisons
☐	☒	A full description of the statistical parameters including central tendency (e.g. means) or other basic estimates (e.g. regression coefficient) AND variation (e.g. standard deviation) or associated estimates of uncertainty (e.g. confidence intervals)
☐	☒	For null hypothesis testing, the test statistic (e.g. *F*, *t*, *r*) with confidence intervals, effect sizes, degrees of freedom and *P* value noted<br>*Give P values as exact values whenever suitable.*
☒	☐	For Bayesian analysis, information on the choice of priors and Markov chain Monte Carlo settings
☒	☐	For hierarchical and complex designs, identification of the appropriate level for tests and full reporting of outcomes
☐	☒	Estimates of effect sizes (e.g. Cohen's *d*, Pearson's *r*), indicating how they were calculated

Our web collection on statistics for biologists contains articles on many of the points above.

Software and code

Policy information about availability of computer code

Data collection	A haptic device (PHANToM Premium 1.5 HF) was programed by C++ (Visual studio version 2008) to be used as a manipulandum to collect the reaching data.
Data analysis	All the data analysis was carried out using Matlab version 2020b (Mathworks). Data frame toolbox (https://www.diedrichsenlab.org/toolboxes/matlab_toolboxes.htm) was used for creating the figures. Code to reproduce the figures to interpret the data in the paper have been deposited on the OSF website (https://osf.io/n7z4q/), along with the data.

For manuscripts utilizing custom algorithms or software that are central to the research but not yet described in published literature, software must be made available to editors and reviewers. We strongly encourage code deposition in a community repository (e.g. GitHub). See the Nature Portfolio guidelines for submitting code & software for further information.

Data

Policy information about availability of data

All manuscripts must include a data availability statement. This statement should provide the following information, where applicable:

- Accession codes, unique identifiers, or web links for publicly available datasets
- A description of any restrictions on data availability
- For clinical datasets or third party data, please ensure that the statement adheres to our policy

All the data required to evaluate the conclusions of the study are presented in the paper and in the Supplementary Materials, and have been deposited on the OSF website (https://osf.io/n7z4q/).

Research involving human participants, their data, or biological material

Policy information about studies with human participants or human data. See also policy information about sex, gender (identity/presentation), and sexual orientation and race, ethnicity and racism.

Reporting on sex and gender
46 female and 101 male volunteers participated in the study (self-reported). Sex and gender were not considered in the study design, since we lack specific hypothesis of any group difference regarding the effect of interest. Therefore, any sex- and gender-based analysis is not performed.

Reporting on race, ethnicity, or other socially relevant groupings
Participants were not classified into different race, ethnicity of other social categories.

Population characteristics
Participants were recruited from the participant pool of CiNet (mainly university students and researchers), ranging in age from 19-38 years old. All subjects were right-handed.

Recruitment
All participants were recruited at CiNet via an online system (SONA systems) for volunteers, for the compensation of 1000JPY/hour for participation.

Ethics oversight
National Institute of Information and Communications Technology (NICT) ethical committee

Note that full information on the approval of the study protocol must also be provided in the manuscript.

Field-specific reporting

Please select the one below that is the best fit for your research. If you are not sure, read the appropriate sections before making your selection.

☒ Life sciences ☐ Behavioural & social sciences ☐ Ecological, evolutionary & environmental sciences

For a reference copy of the document with all sections, see nature.com/documents/nr-reporting-summary-flat.pdf

Life sciences study design

All studies must disclose on these points even when the disclosure is negative.

Sample size
Participants were randomly sampled and assigned to each experiment. We did not pre-define the sample size. While sample sizes for experiment groups in analogous motor learning studies typically range between 8 and 12, here, we used larger sample sizes in Experiment 1 (N = 19 each for both the certain and uncertain condition) due to the cross-participant design, and also to account for the possible noise induced by the trial-by-trial fluctuation of the subjective uncertainty level within participants. To ensure a similar level of effect size as in Experiment 1, we used a similar number of participants in the rest of the experiments (Experiment 2-1, n=19; 2-2, n=20; 2-3, n=17; 3-1, n=18; 3-2, n=16; 3-3, n=15; 4, n=18; 5, n=14)

Data exclusions
In each experiment, trials were excluded if the 1) reaction times (movement onset concerning the visual stimulus onset) were too fast (<100 ms; likely not judging the stimulus) or too slow (1,500 ms>; judging after the stimulus disappearance), 2) did not reach properly to the target (<75% of the maximum distance), and when the movement direction reversed after going 2.5 cm to the opposite direction before reaching to the target. If the trial exclusion rate exceeded 30% of the data in the last block of the learning phase or the retrieval/test phase, the participants were excluded from further analysis. Furthermore, if the overall choice rate during the retrieval phase was biased towards one direction (>70%) (e.g., moving [making a decision] to the right in most of the trials), the participant was also excluded because of the asymmetrical motor learning experience between the two directions. See the method section below for task details. Note that these exclusion criteria were set to exclude data/participants who did not follow the instructions of the experiments and maintain the same data quality across participants. However, including excluded participants in the analysis did not qualitatively change the results.
Based on the above criteria, in Experiment 1, three participants from each certain and uncertain group were excluded. Likewise, two participants were excluded from the analysis of Experiment 2-3, 2-4, and 3, respectively.

Replication
Result of Experiment 1 was conceptually replicated in Experiment 2-1 and 2-2. Experiment 3 also included the replication of Experiment 1.

Randomization
Participants were randomly assigned to each experiment.

Blinding	Experimenter was not blind to the purpose of the experiment. However, the experimenter could not always monitor the condition of the current trial, due to the location they sat during the experiment. Furthermore, the effect was also replicated (Experiments 3-1, 3-2) when different experimenter who were blind to the purpose of the study conducted the experiment.

Reporting for specific materials, systems and methods

We require information from authors about some types of materials, experimental systems and methods used in many studies. Here, indicate whether each material, system or method listed is relevant to your study. If you are not sure if a list item applies to your research, read the appropriate section before selecting a response.

Materials & experimental systems

n/a	Involved in the study	
☒	☐	Antibodies
☒	☐	Eukaryotic cell lines
☒	☐	Palaeontology and archaeology
☒	☐	Animals and other organisms
☒	☐	Clinical data
☒	☐	Dual use research of concern
☒	☐	Plants

Methods

n/a	Involved in the study	
☒	☐	ChIP-seq
☒	☐	Flow cytometry
☒	☐	MRI-based neuroimaging
